# Effects of Fermented Pea–Wheat Ingredient Inclusion in Soybean Meal-Replacement Diets on Intestinal Adaptation, Gut Microbiota, and Fecal Consistency in Weaned Piglets

**DOI:** 10.3390/ani16101526

**Published:** 2026-05-16

**Authors:** Botond Alpár, László Varga, Alex Váradi, Eszter Kaszab, Zoltán Somogyi, Tamás Tóth

**Affiliations:** 1 Wittmann Antal Multidisciplinary Doctoral School of Plant, Animal, and Food Sciences, Széchenyi István University, 2 Vár Square, 9200 Mosonmagyaróvár, Hungary; botond.alpar@agrofeed.hu; 2 Department of Food Science, Széchenyi István University, 15-17 Lucsony Street, 9200 Mosonmagyaróvár, Hungary; 3 Department of Bioinformatics, One Health Institute, Faculty of Health Sciences, University of Debrecen, 98 Nagyerdei Blvd., 4032 Debrecen, Hungary; varadi.alex@pte.hu (A.V.); kaszab.eszter@univet.hu (E.K.); 4 Institute for Translational Medicine, Medical School, University of Pécs, 12 Szigeti Street, 7624 Pécs, Hungary; 5 Department of Microbiology and Infectious Diseases, University of Veterinary Medicine Budapest, 23-25 Hungária Blvd., 1143 Budapest, Hungary; 6 National Laboratory for Infectious Animal Diseases, Antimicrobial Resistance, Veterinary Public Health and Food Chain Safety, 2 István Street, 1078 Budapest, Hungary; somzol92@gmail.com; 7 Department of Pharmacology and Toxicology, University of Veterinary Medicine, 2 István Street, 1078 Budapest, Hungary; 8 Agricultural and Food Research Center, Széchenyi István University, 1 Egyetem Square, 9026 Győr, Hungary; toth.tamas@sze.hu

**Keywords:** fermentation, field peas, gut microbiota, intestinal morphology, weaned piglets

## Abstract

Weaning disrupts intestinal structure and function in piglets and frequently results in post-weaning diarrhea, highlighting the need for effective nutritional alternatives to medicinal feed additives. This study evaluated non-fermented field peas and diets containing different inclusion levels of a fermented pea–wheat ingredient as substitutes for soybean meal in restricted liquid diets for weaned piglets. Fifty-six piglets were assigned to four dietary treatments for 14 days after weaning: non-fermented soybean meal, non-fermented peas, partial inclusion of the fermented pea–wheat ingredient, or full inclusion of the fermented pea–wheat ingredient. Fecal consistency, plasma inflammatory cytokines, small-intestinal morphology, crypt cell proliferation, and intestinal microbiota composition were assessed. Plasma interleukin-1β, interleukin-6, and tumor necrosis factor-α remained low and did not differ among treatments, indicating no measurable systemic inflammatory response. Diets containing the fermented pea–wheat ingredient were associated with differences in early intestinal morphology, particularly villus height on day 4 after weaning. Treatment- and segment-specific variation was also observed in crypt cell proliferation. Microbiota analysis showed differences in community composition among treatments, with diets containing the fermented pea–wheat ingredient associated with a higher relative abundance of lactic acid-producing genera. At the pen level, full inclusion of the fermented pea–wheat ingredient was consistently associated with low fecal scores, whereas partial inclusion was associated with higher fecal scores throughout the study period. Overall, these findings suggest that, under the controlled conditions of this study, diets containing the full inclusion level of the fermented pea–wheat ingredient were associated with indicators of intestinal adaptation and consistently low pen-level fecal scores. Further studies with replicated pen designs, longer-term performance evaluation, and functional analyses are needed before practical recommendations can be made.

## Simple Summary

Weaning is a critical stage in pig production because young pigs often experience digestive disturbances and diarrhea when transitioning from milk to solid feed. At the same time, producers need safe and natural feeding strategies, as some traditional feed additives, such as pharmacological levels of zinc oxide and prophylactic antibiotics, are no longer permitted in the European Union. This study evaluated non-fermented field peas and diets containing different inclusion levels of a fermented pea–wheat ingredient as alternatives to soybean meal in liquid diets for newly weaned piglets. Piglets received diets containing soybean meal, non-fermented peas, partial inclusion of the fermented pea–wheat ingredient, or full inclusion of the fermented pea–wheat ingredient. Full inclusion of the fermented pea–wheat ingredient was associated with consistently low pen-level fecal scores, differences in early small-intestinal morphology, and variation in gut microbial composition. Blood analyses indicated that none of the diets resulted in detectable systemic inflammatory responses. Partial inclusion of the fermented pea–wheat ingredient was also associated with changes in intestinal development; however, higher pen-level fecal scores were observed during the study period. Overall, these findings suggest that the level of fermented pea–wheat ingredient inclusion may influence the response to pea-based diets. Under the conditions tested, diets containing the full inclusion level of the fermented pea–wheat ingredient may warrant further investigation as a dietary strategy for weaned piglets.

## 1. Introduction

There are two critical periods in piglet production: the first few days after birth and the approximately 2-week period immediately after weaning. Weaning, environmental change, and diet composition directly affect gastrointestinal development in piglets [1,2] and indirectly modulate the gut microbiome [3]. Piglets are particularly susceptible to enteric pathogens during this period, and post-weaning diarrhea is the most common consequence. Post-weaning diarrhea typically occurs after a latency period of 3–4 days and peaks around 1 week after weaning [4]. For many years, zinc oxide was the main strategy used to prevent digestive disorders and the associated diarrhea in weaned piglets. More recently, however, the European Union prohibited the use of therapeutic doses of zinc oxide and prophylactic antibiotics in feed, creating a major challenge for the pig sector. As a result, natural feeding strategies capable of improving intestinal health and suppressing pathogen proliferation have received increasing attention in young pig nutrition. These strategies include practical feeding approaches [5], specialized feed formulation techniques, and the use of alternative raw materials and feed additives derived from natural sources, including plants, minerals, and microorganisms [6,7,8].

One of these emerging approaches is feed fermentation, which offers both nutritional and physiological benefits for pigs. Controlled fermentation with lactic acid bacteria (LAB) is particularly promising [9] because the microorganisms partially predigest the feed substrate. The resulting reduction in particle size increases the surface area available for digestive enzymes, thereby improving nutrient utilization in the gastrointestinal tract. In addition, the proliferation of beneficial bacteria, the formation of bioactive compounds such as bacteriocins and enzymes, the production of lactic acid, and the resulting decrease in pH can all positively affect intestinal microbiota composition and help maintain microbial balance [10]. These changes may reduce digestive disorders, improve digestive efficiency, and support immune function [11,12,13]. Previous studies have also shown that fermentation can reduce antinutritional factors [14] such as phytates and protease inhibitors, which otherwise impair nutrient absorption [15]. These changes are especially relevant in weaned piglets, which are highly prone to enteritis and digestive disturbances during this period.

Liquid feeding makes it possible to ferment either the entire feed or selected feed components [16], and this approach has become increasingly common in pig production. In practice, partial feed fermentation has gained more widespread acceptance than complete feed fermentation, and fermentation of grain fractions in particular has been associated with improved feed characteristics. More recently, combinations of cereals with protein sources and/or industrial by-products have also been used, allowing fermented ingredients to be incorporated flexibly into multiphase feeding systems [17].

LAB-fermented cereal fractions, such as wheat and barley, have been reported to improve gut morphology, contribute to villus development, increase the villus-to-crypt ratio [18], modulate fecal microbiota composition, and downregulate pro-inflammatory cytokines in weaned piglets [19]. Among fermented protein sources, fermented soybean products are the most extensively studied. Fermented soybean meal has been shown to modulate microbiota structure, improve small-intestinal morphology, reduce inflammatory cytokine levels, and support overall gut health [20,21,22]. Lactic acid fermentation can also reduce trypsin inhibitor activity and increase protein hydrolysis [23].

Replacing imported soybean meal with regionally produced protein crops could provide economic and environmental benefits, reducing the ecological footprint of pig farming and supporting more sustainable agricultural practices. In this context, grain legumes such as field peas (*Pisum sativum* L.) represent a promising alternative protein source for pigs due to their favorable nutritional composition and capacity to replace conventional protein ingredients [24]. In addition to their protein content, legumes can supply fermentable carbohydrates, thereby influencing digestive processes and intestinal fermentation patterns. Furthermore, using locally produced legumes has been recognized as an important strategy for reducing dependence on imported soybean meal and enhancing the sustainability of livestock production systems [25].

However, the use of grain legumes in piglet nutrition is limited by the presence of antinutritional factors and variability in nutrient digestibility, particularly during the post-weaning period [26]. In addition to ingredient selection and processing strategies, dietary formulation approaches are commonly employed to support intestinal function during the post-weaning period. Low-crude-protein diets are often used to minimize the amount of undigested protein that reaches the large intestine, thus reducing harmful protein fermentation and lowering the risk of post-weaning diarrhea [8,27,28,29,30]. Current recommendations emphasize reducing crude protein while maintaining an adequate supply of essential amino acids [5]. Furthermore, the inclusion of insoluble fiber has been reported to affect digesta passage and intestinal conditions during the early post-weaning period [31,32]. Processing strategies such as fermentation have therefore been investigated to improve the nutritional value of plant-based feed ingredients by enhancing digestibility and reducing antinutritional compounds [13,33,34]. In piglets, fermented plant protein sources such as rapeseed meal and alfalfa have been reported to influence intestinal microbial populations and gut morphology [35,36] and have been proposed as a dietary approach to reduce reliance on pharmacological levels of zinc oxide [37]. However, the magnitude and consistency of these effects vary across studies.

Despite these advances, information on fermented field peas remains limited, and it is unclear whether different degrees of fermentation may result in distinct biological responses in weaned piglets.

Although fermentation is widely regarded as an effective strategy for enhancing the nutritional and functional properties of plant-based feed ingredients, its impact is not always consistent and can vary depending on the extent of fermentation and the resulting biochemical modifications of the substrate. The degree of antinutritional factor degradation, microbial metabolite formation, and changes in nutrient availability may differ among diets containing different levels of fermented pea ingredients, potentially leading to distinct biological responses. These differences may, in turn, influence digestive processes, intestinal morphology, and microbial colonization in different ways. However, these aspects have received limited attention in the context of field peas, particularly in weaned piglets, whose gastrointestinal tract is highly sensitive to dietary interventions.

Therefore, the present study aimed to evaluate non-fermented field peas and different inclusion levels of a fermented pea–wheat ingredient as substitutes for soybean meal in restricted liquid diets for weaned piglets. Specifically, we investigated intestinal adaptation, including small-intestinal morphology, crypt cell proliferation, gut microbiota composition at the small intestine–cecum interface, and selected blood parameters, with emphasis on inflammatory markers. All measurements were performed under restricted feeding conditions. It was hypothesized that diets differing in the inclusion level of the fermented pea–wheat ingredient would be associated with distinct gastrointestinal responses.

## 2. Materials and Methods

### 2.1. Animals and Experimental Design

**Figure 1:**
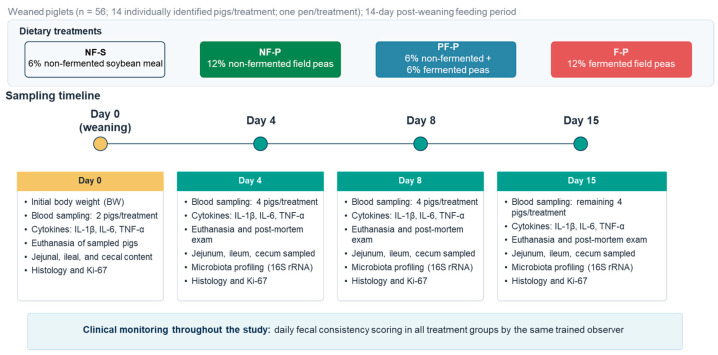
Experimental design: treatments, sampling timeline, and analytical endpoints.

A total of 56 freshly weaned commercial Danbred piglets (Duroc × Landrace × Yorkshire), 31 days of age [standard deviation (SD) = 1.0 day] and with specific pathogen-free health status, were included in the study (Figure 1). Mean initial body weight was 9.37 kg (SD = 1.08). During the suckling period, piglets had free access to water and creep feed. Male piglets were castrated on day 3 after birth. At the farm of origin, piglets were selected as follows: from each of 14 sows, four piglets of similar size and sex were chosen on the basis of physical examination and marked with four different colored ear tags (white, green, blue, or red). On the day of weaning, sows were removed from the piglets at 7:30 a.m., after which the 56 experimental piglets were transferred to the Experimental Farm of the University of Veterinary Medicine Budapest.

At the start of the experiment, all piglets were identified individually. They were then allocated to four treatment groups (*n* = 14 per treatment; no littermates within the same group) and housed in four identical nursery pens. The piglets were selected from different litters and distributed across the treatments to minimize the potential effects of litter; however, litter was not included as a statistical factor. Dietary treatment during the 14-day post-weaning period was applied at the pen level. Thus, the pen, not the individual piglet, was the experimental unit for the dietary treatment. Because only one pen was available per treatment, the design did not include replicated experimental units for confirmatory treatment-level inference. Individual piglets were considered observational units only for endpoints measured on individual samples, including blood parameters, intestinal morphology, Ki-67 immunostaining, and microbiota composition. Consequently, statistical comparisons based on individual-level data should be interpreted as exploratory and descriptive of the sampled animals within each treatment pen, not as fully replicated pen-level treatment effects.

The experimental room provided optimal environmental conditions for newly weaned piglets, including a programmed light–dark cycle, controlled temperature and humidity, and an enriched environment. Piglets were housed in conventional plastic fully slatted-floor pens (250 cm × 300 cm), each fitted with an additional rubber resting mat (100 cm × 100 cm). Pens were separated by solid plastic partitions to prevent direct physical contact and minimize cross-contamination between treatments. Each pen was equipped with a 100 cm stainless steel trough suitable for liquid feeding and secured to the plastic floor with spring hooks. A cup drinker provided ad libitum access to water throughout the experiment.

### 2.2. Feeding

#### 2.2.1. Diets

Four dietary treatments were used in this study. The first diet (NF-S, white) contained 6% non-fermented soybean meal. In the second diet (NF-P, green), all soybean meal was replaced with 12% non-fermented field peas, matched for protein content. The level of field pea inclusion (12%) was selected to replace 6% of the protein contribution from soybean meal, taking into account the lower crude protein concentration and digestibility differences in peas compared to soybean meal. The diets were formulated to achieve comparable standardized ileal digestible (SID) amino acid and net energy levels across the treatments. This formulation minimized differences in digestible amino acid and energy supply among treatments, although the design did not allow the effects of fermentation to be fully separated from the overall dietary formulation and feeding system. In the third diet (PF-P, blue), 50% of the field pea fraction (6%) was supplied through the fermented pea–wheat ingredient, whereas in the fourth diet (F-P, red), the full field pea fraction (12%) was supplied through the fermented pea–wheat ingredient. The PF-P treatment was designed to represent lower exposure to the fermented pea–wheat ingredient, with only half of the total pea fraction provided in fermented form and the other half remaining non-fermented. By contrast, the entire pea fraction in the F-P treatment was supplied in fermented form. This design enabled us to investigate whether partial or complete replacement with fermented pea–wheat ingredient would elicit different biological responses, providing insight into the potential dose-related effects of inclusion of the fermented pea–wheat ingredient.

All experimental diets were low in crude protein but balanced for essential amino acids, and insoluble fiber was included in the basal formulation. Piglets were fed liquid diets at a water-to-feed ratio of 2.5:1, resulting in a final dry matter content of 25.4%. Diets were formulated using Bestmix for Windows^®^ 3.35 (ADIFO Software) to maintain equivalent SID amino acid and net energy concentrations across treatments. Because both dry ingredients and high-moisture fermented components were used, diets were also standardized on a dry matter basis. All diets were free of antibiotics and pharmacological concentrations of copper and zinc. The composition of the experimental diets is presented in Table 1.

#### 2.2.2. Preparation of the Fermented Feed Ingredient

The fermented feed ingredient (F-75P25W) was prepared by LAB-controlled batch fermentation in liquid phase according to the recommendations of Dr. Ferm (Budapest, Hungary). The fermented pea–wheat ingredient was produced by adding 2.33 kg of warm water (38 °C) to a mixture of 0.75 kg field peas and 0.25 kg wheat, both hammer-milled through a 3 mm sieve. The mixture was inoculated with 0.22 mL of diluted, 24 h-subcultured RS-L Health premixture from Dr. Ferm (*Lactiplantibacillus plantarum* DSM 3676, *Lactiplantibacillus plantarum* DSM 3677, *Enterococcus faecium* DSM 22502; minimum total bacterial count: 2.0 × 10^8^ CFU/g) and fermented for 24 h. The fermented feed ingredient contained peas and wheat in a 3:1 ratio (as-fed basis before hydration), selected based on preliminary optimization of fermentation characteristics. Therefore, the fermented component should be interpreted as a fermented pea–wheat ingredient rather than as fermented peas alone.

The fermented feed ingredient was prepared in a 125 L mini fermenter (WEDA, Lutten, Germany), which agitated the liquid mixture for 1 min every 45 min to ensure homogeneity during fermentation. The final ferment had a dry matter content of 26.3%, a pH of 4.0 (pH-Fix 2.0–9.0 indicator strips; Macherey–Nagel, Düren, Germany), *Lactiplantibacillus plantarum* at 10 log_10_ CFU/kg, and *Enterococcus faecium* at 8 log_10_ CFU/kg.

#### 2.2.3. Feed Distribution

A restricted liquid-feeding regimen was used throughout the study. Piglets were fed according to a predetermined intake curve based on the metabolizable energy recommendations of DanBred International and on observed intake patterns of piglets with similar genetics at the experimental farm. Although the original plan was to increase the daily ration by 25 g, the higher initial body weights of the piglets required larger adjustments on days 3 and 4 (Table 2).

Feed was offered four times daily at 7:00 a.m., 11:00 a.m., 3:00 p.m., and 7:00 p.m., with each daily allotment divided equally across meals. Liquid feed was prepared immediately before feeding according to the number of piglets per pen. This feeding strategy ensured that troughs were emptied between meals, thereby maintaining hygiene and minimizing uncontrolled secondary fermentation.

### 2.3. Sampling and Postmortem Examination

During the veterinary examination after arrival, all piglets were deemed healthy. Individual body weights were recorded at the start of the study. Thereafter, 3 mL of blood was collected from the cranial vena cava from two randomly selected pigs per group for cytokine analysis. These same pigs also underwent postmortem examination after euthanasia. Euthanasia was performed by rapid intravenous administration of pentobarbital (100 mg/kg) into the lateral auricular vein of the left ear using a 23G, ¼-inch needle, thereby minimizing animal suffering.

The general condition of the animals was assessed during pathological examination, and no external lesions were detected. The condition of the internal organs was consistent with the animals’ age and stage of development. Examination of the gastrointestinal tract focused on the intestines and stomach, including the esophageal entrance region. No gross lesions were observed, and therefore no additional pathological investigations were performed beyond those defined in the study objectives. After gastrointestinal examination, intestinal contents were collected from the jejunum, ileum, and cecum for microbiota analysis, followed by tissue sampling from the same regions for histopathological evaluation. The jejunum, ileum, and cecum were sampled for microbiota and histological analyses to assess region-specific responses.

On study days 4 and 8, blood samples were collected from four pigs per group; on study day 15, samples were collected from the remaining four pigs per group. After euthanasia, postmortem examination was performed and intestinal contents and tissue samples were collected from the jejunum, ileum, and cecum. Blood collection and euthanasia were performed in the same manner as on day 0.

### 2.4. Observations

#### 2.4.1. Fecal Consistency Scoring

Throughout the experimental period, fecal consistency in the four treatment groups was assessed daily by the same trained observer using a previously described four-category scoring scale [38,39]. The categories were defined as follows: 0 = normal/firm feces, 1 = soft feces, 2 = mild/loose diarrhea, and 3 = severe/watery diarrhea. Observer blinding was not feasible under these conditions. Photographs were also taken for each treatment to aid differentiation among consistency categories.

Because fecal consistency was recorded at the pen level and each dietary treatment was represented by one pen, the pen was the experimental unit for this endpoint. Therefore, fecal consistency data were not subjected to inferential treatment comparisons and are reported descriptively.

#### 2.4.2. Plasma Inflammatory Cytokines (IL-1β, IL-6, and TNF-α)

Plasma concentrations of inflammatory cytokines were determined using a sandwich ELISA, following the manufacturer’s instructions. Pig-specific ELISA kits (Merck, Darmstadt, Germany) were used to quantify interleukin-1β (IL-1β), interleukin-6 (IL-6), and tumor necrosis factor-α (TNF-α).

Absorbance was measured at 450 nm using a SpectraMax ID3 spectrophotometer (Molecular Devices, San Jose, CA, USA) and calibration curves were generated using the provided standards. However, preliminary evaluation of the data indicated that many of the measured cytokine concentrations were close to or below the lower limits of quantification (i.e., IL-1β: 6 pg/mL; IL-6: 45 pg/mL; TNF-α: 20 pg/mL). Under these conditions, converting absorbance values to concentrations could introduce substantial uncertainty. Therefore, background-corrected absorbance values were used for comparative statistical analysis among treatments, and the results were interpreted as indicative of relative differences rather than absolute cytokine concentrations.

Statistical analyses were performed in R 4.2.2 (R Foundation for Statistical Computing, Vienna, Austria). For each sampling day, background-corrected absorbance values were compared among dietary treatments using one-way ANOVA, followed by Tukey’s post hoc test. However, these analyses used the individual sampled piglet as the observational unit, and because dietary treatment was not replicated at the pen level, the resulting *p*-values should be regarded as exploratory and should not be interpreted as confirmatory evidence of replicated dietary treatment effects.

#### 2.4.3. Gut Microbiota Composition of Jejunal, Ileal, and Cecal Samples

##### Nucleic Acid Isolation

Nucleic acids were extracted from intestinal content samples collected from the jejunum, ileum, and cecum using the ZymoBIOMICS™ DNA Miniprep Kit (Zymo Research, Tustin, CA, USA) according to the manufacturer’s instructions.

##### Sequencing

DNA libraries for next-generation sequencing were prepared using the Illumina^®^ Nextera XT DNA Library Preparation Kit (96 samples). First, the V3–V4 hypervariable region of the bacterial 16S rRNA gene was amplified from frozen DNA samples using the primer pair 16SBact-0341 Fwd (5′ TCGTCGGCAGCGTCAGATGTGTATAAGAGACAGCCTACGGGNGGCWGCAG) and 16SBact-0785 Rev (5′ GTCTCGTGGGCTCGGAGATGTGTATAAGAGACAGGACTACHVGGGTATCTAATCC). Amplicons were purified using AMPure XP magnetic beads (Beckman Coulter, Brea, CA, USA). Purified DNA fragments were diluted to 0.5 ng/µL and individually indexed using the Nextera XT Index Kit v2 Set D (Illumina, San Diego, CA, USA), with one i5 and one i7 index per sample, according to the manufacturer’s instructions.

The exact DNA concentration of indexed amplicons was determined using the Qubit^®^ dsDNA HS Assay (Thermo Fisher Scientific, Waltham, MA, USA), and average fragment length was measured with an Agilent Bioanalyzer (Agilent Technologies, Santa Clara, CA, USA). Molar concentration was calculated as follows:

Molarity (nM) = (ng/µL × 10^6^)/(660 g/mol × average library size [bp])

Each sample was diluted to 4 nM, and 20 µL of each was combined to prepare the final library pool. Samples that did not reach 4 nM after purification were added to the pool undiluted. Pool concentration was rechecked using the Qubit^®^ dsDNA HS Assay (Thermo Fisher Scientific) and adjusted to 4 nM if necessary. Subsequently, 5 µL of the DNA pool was denatured with an equal volume of 0.2 M NaOH for 5 min and neutralized with 990 µL HT1 buffer. Similarly, 5 µL of 4 nM PhiX solution (PhiX Control v3, Illumina) was denatured. The final sequencing library was adjusted to 6 pM with 35% PhiX as follows: 117 µL denatured DNA pool + 63 µL denatured PhiX + 420 µL HT1 buffer. Sequencing was performed on an Illumina MiSeq platform using the MiSeq Reagent Kit v3 (600 cycles).

##### Bioinformatics Analysis

After sequencing, raw reads were quality-filtered and trimmed using fastp according to a standard quality-control pipeline. During this step, adapter and primer sequences were removed from the ends of reads, and low-quality reads were discarded. Subsequently, unique amplicon sequence variants (ASVs) were inferred and taxonomically assigned using the dada2 pipeline.

After processing, the median ASV count per sample (*n* = 165) was 19,542, with a range of 19 to 114,318. To reduce uncertainty associated with low sequencing depth, samples with fewer than 1500 reads (*n* = 6) were excluded from the microbiome analysis. This cut-off was selected to retain as many samples as possible while excluding those with the greatest uncertainty.

Alpha diversity was evaluated using the Shannon and inverse Simpson indices, calculated with the diversity function in the vegan package (v2.6-10) in R v4.3.2 (R Foundation for Statistical Computing). For consistency with the genus-level compositional analyses, ASV counts were aggregated at the genus level prior to diversity calculations. This approach was used to improve interpretability and reduce the influence of low-abundance taxa and sequencing variability, although it reduced taxonomic resolution. Group differences were assessed according to treatment, sampling day (days 4, 8, and 15), and intestinal region (small intestine and cecum). Statistical significance was tested using the Kruskal–Wallis test followed by Dunn’s post hoc test where appropriate. Results were visualized using boxplots.

For microbiota analyses, each intestinal-content sample from an individual piglet was treated as an observational unit. Because all piglets within a treatment shared the same pen, these analyses cannot separate dietary treatment effects from potential pen effects and are therefore interpreted as exploratory.

Relative taxonomic composition at the genus level was calculated by normalizing counts within each sample. Taxa with a mean relative abundance below 2% were grouped as “Other.” Microbiota composition was evaluated both at the level of individual samples and as treatment averages at the genus level. Stacked bar plots were generated to visualize relative abundance across treatments, sampling days, and intestinal regions.

Community structure was assessed by non-metric multidimensional scaling (NMDS) based on Bray–Curtis dissimilarity using the metaMDS function in the vegan package. NMDS ordinations were linked to metadata, including treatment, intestinal region, and sampling day. Group separation was further illustrated using 90% confidence ellipses around treatment centroids.

#### 2.4.4. Intestinal Histopathology

##### Hematoxylin–Eosin Staining

**Figure 2:**
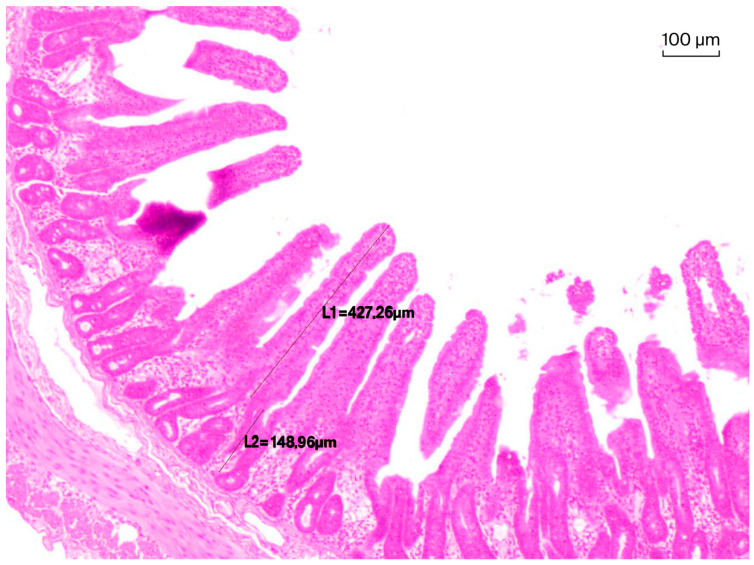
Representative measurements of villus height and crypt depth.

During sampling, approximately 2 cm segments were excised. If necessary, the mucosal surface was gently rinsed under running water before fixation in 10% neutral buffered formaldehyde. For histological processing, three sections from each intestinal sample were placed into an embedding cassette. After routine paraffin embedding and hematoxylin–eosin staining, samples were examined by light microscopy to determine villus height (µm), crypt depth (µm), and the villus height-to-crypt depth ratio. Measurements were performed using an Olympus CX41 microscope equipped with a VSI RZ502 camera and ImageView software, version X64 (Olympus Corporation, Tokyo, Japan) at 40× magnification. Representative measurements of villus height and crypt depth are shown in Figure 2.

In all cases, only properly oriented villi, defined as straight and perpendicular to the basement membrane, were measured. Villus height was measured from the tip of the villus to the shoulder of the adjacent crypt, and crypt depth was measured from this point to the basement membrane. For each excision, eight villi and their corresponding crypts were measured, yielding 24 paired measurements per intestinal segment. In total, 8064 measurements were obtained. Mean villus height and mean crypt depth were then calculated for each intestinal segment within each animal and used for statistical analysis.

##### Statistical Analysis

Villus height, crypt depth, and the villus height-to-crypt depth ratio were analyzed to determine the effects of dietary treatment, intestinal segment, sampling day, and their interactions. Samples were collected from the duodenum, jejunum, and ileum on days 0, 4, 8, and 15 after weaning in piglets fed NF-S, NF-P, PF-P, or F-P diets. Data distribution was assessed using the Kolmogorov–Smirnov and Shapiro–Wilk tests. Statistical analyses were performed using a univariate general linear model (GLM), with diet, intestinal segment, sampling day, and their interactions included in the model. When significant effects were detected, means were compared using Tukey’s post hoc test. Statistical significance was set at *p* < 0.05. All analyses were performed using SPSS 26.0 for Windows (IBM, Armonk, NY, USA).

For these histological measurements, values were averaged at the individual-animal level and the individual piglet was used as the observational unit. Nevertheless, as dietary treatment was applied to one pen per treatment, these comparisons should be interpreted as exploratory treatment-associated differences rather than confirmatory pen-replicated treatment effects.

##### Immunohistochemistry

**Figure 3:**
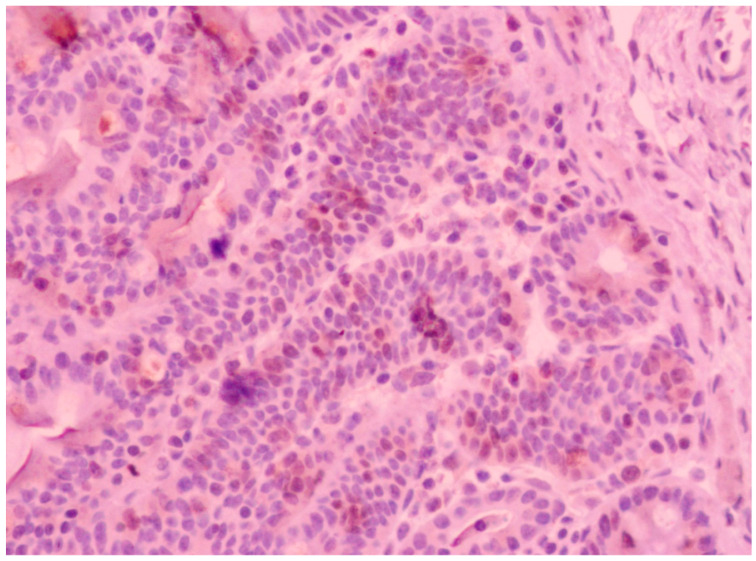
Representative Ki-67 immunostaining of intestinal crypt cells.

To assess crypt cell proliferation, Ki-67 immunostaining was performed on the same intestinal sections. After appropriate pretreatment, proliferating cell nuclei were detected using Monoclonal Mouse Anti-Human Ki-67 Antigen, clone MIB-1 (Agilent Technologies). The number of positively stained epithelial nuclei was determined by counting strongly stained epithelial cells in three consecutive fields at 200× magnification. Cells located within the crypt lumen or lamina propria were excluded. In total, nine measurements were performed per intestinal section, with approximately 30–110 cells counted per measurement and more than 30,000 cells counted overall. Data were averaged at the animal level for each intestinal section and used for statistical analysis. A representative Ki-67 staining pattern is shown in Figure 3.

##### Statistical Analysis

The Ki-67 proliferation index was determined in the duodenum, jejunum, and ileum on days 4, 8, and 15 after weaning and was expressed as the percentage of Ki-67-positive nuclei relative to the total number of epithelial nuclei counted. Data distribution was assessed using the Kolmogorov–Smirnov and Shapiro–Wilk tests. Statistical analysis was performed using a univariate GLM including dietary treatment, intestinal segment, sampling day, and their interactions as fixed effects. When significant effects were detected, means were compared using Tukey’s post hoc test. Statistical significance was set at *p* < 0.05. All analyses were performed using SPSS 26.0 for Windows (IBM). As with the other individually sampled endpoints, this analytical choice does not provide independent pen-level replication of the dietary treatments; therefore, the Ki-67 results are interpreted as exploratory.

## 3. Results

### 3.1. Fecal Consistency Scoring

The pattern of fecal consistency differed among the treatment groups. Because each dietary treatment was represented by a single pen, fecal consistency data were interpreted descriptively. The F-P pen showed consistently low fecal scores throughout the experimental period, whereas higher scores were observed intermittently in the NF-P pen and more persistently in the PF-P pen (Table 3).

### 3.2. Plasma Cytokines

Plasma cytokine concentrations were generally very low across all dietary treatments. After background correction using blank samples, many values were close to or below the lower limits of quantification of the ELISA assays (i.e., IL-1β: 6 pg/mL; IL-6: 45 pg/mL; and TNF-α: 20 pg/mL). Under these conditions, the cytokine data indicated the absence of detectable systemic inflammatory activation in the experimental piglets (Figures S1–S3).

### 3.3. Associations Between Dietary Treatment and Intestinal Morphology

Villus height, crypt depth, and the villus-to-crypt ratio in the duodenum, jejunum, and ileum on days 0, 4, 8, and 15 after weaning are shown in Table 4. No significant differences among treatments were observed on the day of weaning. By day 4 after weaning, however, piglets receiving diets containing the fermented pea–wheat ingredient (PF-P and F-P) had significantly greater (*p* < 0.001) villus height in all three intestinal segments than piglets receiving non-fermented diets. Regarding the villus-to-crypt ratio, PF-P piglets showed a significantly higher (*p* = 0.002) value only in the duodenum, whereas in the jejunum, the NF-S treatment had the highest value (*p* < 0.001).

On day 8 after weaning, villus height in the duodenum remained significantly greater (*p* < 0.001) in both treatments containing the fermented pea–wheat ingredient (PF-P and F-P). In the jejunum, F-P and NF-P showed the highest values (*p* < 0.001), while in the ileum, NF-P had the highest villus height (*p* = 0.003). For the villus-to-crypt ratio, NF-P was highest in the duodenum (*p* < 0.001), whereas in the jejunum, F-P, NF-S, and PF-P showed the highest values (*p* < 0.001) in descending order.

By day 15 after weaning, villus height remained significantly higher (*p* = 0.004) in PF-P and F-P in the duodenum, in NF-S, NF-P, and PF-P in the jejunum (*p* = 0.047), and in PF-P in the ileum (*p* < 0.001). A significantly higher villus-to-crypt ratio was observed only in the jejunum of the NF-P group.

### 3.4. Ki-67 Proliferation Index

Ki-67-positive crypt cell proliferation was significantly influenced by diet and sampling day, and the responses differed among intestinal segments.

**Figure 4:**
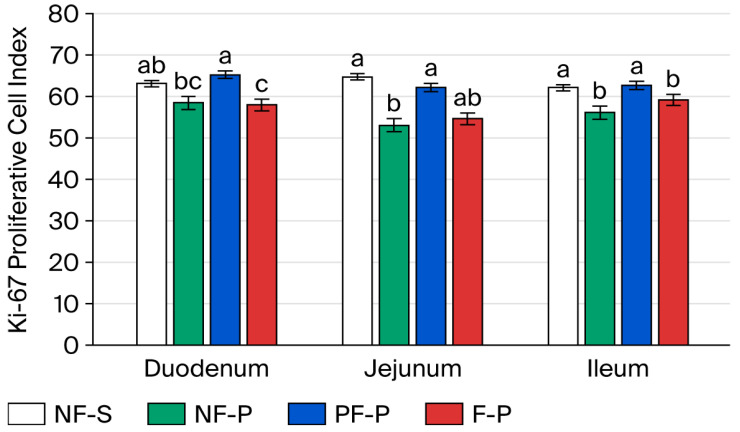
Ki-67 proliferative activity in intestinal crypt cells on day 4 after weaning. Notes: Different letters within intestinal region (i.e., duodenum, jejunum, or ileum) indicate significant differences at *p* < 0.05. NF-S: diet containing 6% non-fermented soybean meal; NF-P: diet in which all soybean meal was replaced by 12% non-fermented field peas; PF-P: diet in which 50% of the field pea fraction (6%) was supplied through the fermented pea–wheat ingredient; F-P: diet in which the full field pea fraction (12%) was supplied through the fermented pea–wheat ingredient.

On day 4 after weaning, piglets fed the PF-P diet showed the highest proliferation index in the duodenum, whereas those fed the F-P diet showed the lowest values (*p* < 0.05; Figure 4). The NF-S and NF-P groups showed intermediate values. In the jejunum, higher proliferative activity was observed in the NF-S and PF-P groups, whereas the NF-P group showed significantly lower Ki-67 values. A similar pattern was observed in the ileum, where NF-S and PF-P were associated with higher proliferation indices, while NF-P and F-P showed lower values (*p* < 0.05).

**Figure 5:**
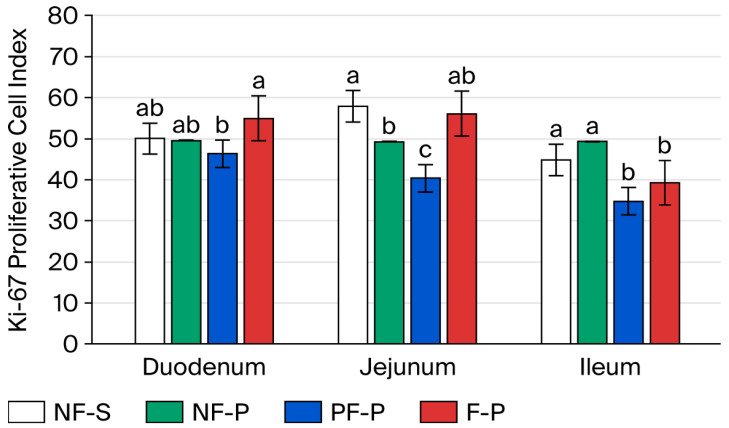
Ki-67 proliferative activity in intestinal crypt cells on day 8 after weaning. Notes: Different letters within intestinal region (i.e., duodenum, jejunum, or ileum) indicate significant differences at *p* < 0.05. NF-S: diet containing 6% non-fermented soybean meal; NF-P: diet in which all soybean meal was replaced by 12% non-fermented field peas; PF-P: diet in which 50% of the field pea fraction (6%) was supplied through the fermented pea–wheat ingredient; F-P: diet in which the full field pea fraction (12%) was supplied through the fermented pea–wheat ingredient.

On day 8 after weaning, the F-P diet increased Ki-67 expression in the duodenum compared with the PF-P diet (*p* < 0.05), whereas the NF-S and NF-P groups again showed intermediate values (Figure 5). The most pronounced treatment effects were observed in the jejunum, where the F-P group showed the highest proliferation index and the PF-P group the lowest (*p* < 0.05). In the ileum, higher proliferative activity was observed in the NF-S and F-P groups, whereas the NF-P and PF-P groups showed lower indices.

**Figure 6:**
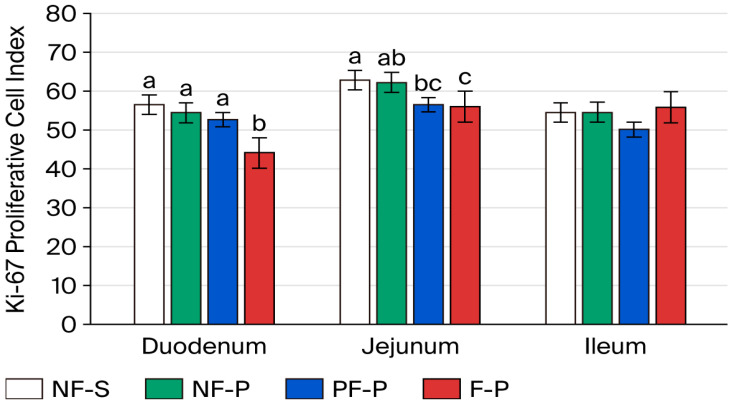
Ki-67 proliferative activity in intestinal crypt cells on day 15 after weaning. Notes: Different letters within intestinal region (i.e., duodenum, jejunum, or ileum) indicate significant differences at *p* < 0.05. NF-S: diet containing 6% non-fermented soybean meal; NF-P: diet in which all soybean meal was replaced by 12% non-fermented field peas; PF-P: diet in which 50% of the field pea fraction (6%) was supplied through the fermented pea–wheat ingredient; F-P: diet in which the full field pea fraction (12%) was supplied through the fermented pea–wheat ingredient.

By day 15, proliferative activity had converged among treatments in all intestinal segments, although some differences persisted. In the duodenum, the NF-S and NF-P groups maintained higher Ki-67 values than the F-P group (*p* < 0.05). In the jejunum, NF-S and NF-P again showed the highest proliferation indices, followed by PF-P, whereas F-P exhibited the lowest value (Figure 6). In the ileum, only minor differences remained among diets.

### 3.5. Development of the Small-Intestinal and Cecal Microbiota

Across all treatments, the intestinal microbiota of weaned piglets was dominated by Firmicutes, with *Faecalibacterium* and lactobacilli-related genera among the most abundant taxa. Nevertheless, differences in microbiota composition were observed among treatments, particularly between the non-fermented diets (NF-S and NF-P) and the diets containing the fermented pea–wheat ingredient (PF-P and F-P). Because PF-P and F-P showed broadly similar microbiota-related trends, selected descriptive comparisons are discussed jointly where appropriate; however, treatment-specific differences were retained in the primary analyses.

#### 3.5.1. Small Intestine (Jejunum and Ileum)

In piglets receiving the NF-S and NF-P diets, the small-intestinal microbiota showed a relatively uniform composition across both intestinal regions and all sampling days (days 4, 8, and 15 after weaning). These communities were consistently dominated by *Faecalibacterium*, with smaller but stable contributions from *Streptococcus*, *Prevotella_9*, *Clostridium sensu stricto 1*, and *Turicibacter*. Only minor temporal shifts were observed, suggesting that the non-fermented diets were associated with a relatively stable and homogeneous small-intestinal microbiota during the early post-weaning period.

In contrast, piglets fed the diets containing the fermented pea–wheat ingredient exhibited a more variable and temporally dynamic small-intestinal microbiota. Although *Faecalibacterium* remained a major genus, the relative abundance of several other genera increased, particularly *Lactobacillus*, *Ligilactobacillus*, *Limosilactobacillus*, *Pediococcus*, *Phascolarctobacterium*, *Prevotella_9*, and *Treponema*. These genera were detected consistently in both the jejunum and ileum. Temporal divergence became more pronounced by day 15, with piglets fed diets containing the fermented pea–wheat ingredient showing an increasing contribution of multiple genera and, consequently, a more even microbial composition than those in the non-fermented groups.

#### 3.5.2. Cecum

Across all treatments, the cecal microbiota was more complex than that of the small intestine. In piglets receiving the NF-S and NF-P diets, *Faecalibacterium* remained the dominant genus on days 4, 8, and 15, with moderate but relatively consistent contributions from *Prevotella_9*, *Subdoligranulum*, and *Streptococcus*. These profiles remained relatively stable over time, mirroring the temporal stability observed in the small intestine.

In contrast, piglets fed diets containing the fermented pea–wheat ingredient showed a more variable cecal microbiota and more pronounced temporal shifts. In addition to *Faecalibacterium*, substantial and fluctuating contributions from *Prevotella_9*, *Phascolarctobacterium*, *Shuttleworthia*, lactobacilli-related genera, and other low-abundance taxa were observed. By day 15, the fermented pea–wheat diets were associated with the greatest variability in cecal community composition, suggesting an association with a broader range of bacterial genera involved in fermentation and carbohydrate utilization in the hindgut.

#### 3.5.3. Changes in Alpha Diversity (Shannon and Inverse Simpson Indices)

**Figure 7:**
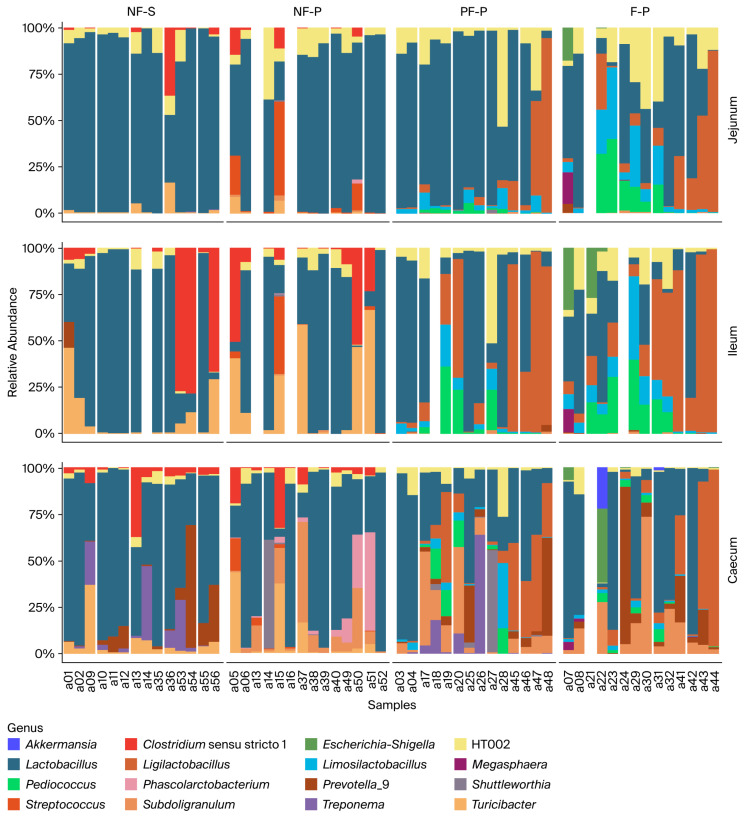
Relative genus-level composition of the jejunal, ileal, and cecal microbiota in weaned piglets fed soybean meal- or pea-based diets differing in fermentation status. Notes: NF-S: diet containing 6% non-fermented soybean meal; NF-P: diet in which all soybean meal was replaced by 12% non-fermented field peas; PF-P: diet in which 50% of the field pea fraction (6%) was supplied through the fermented pea–wheat ingredient; F-P: diet in which the full field pea fraction (12%) was supplied through the fermented pea–wheat ingredient.

**Figure 8:**
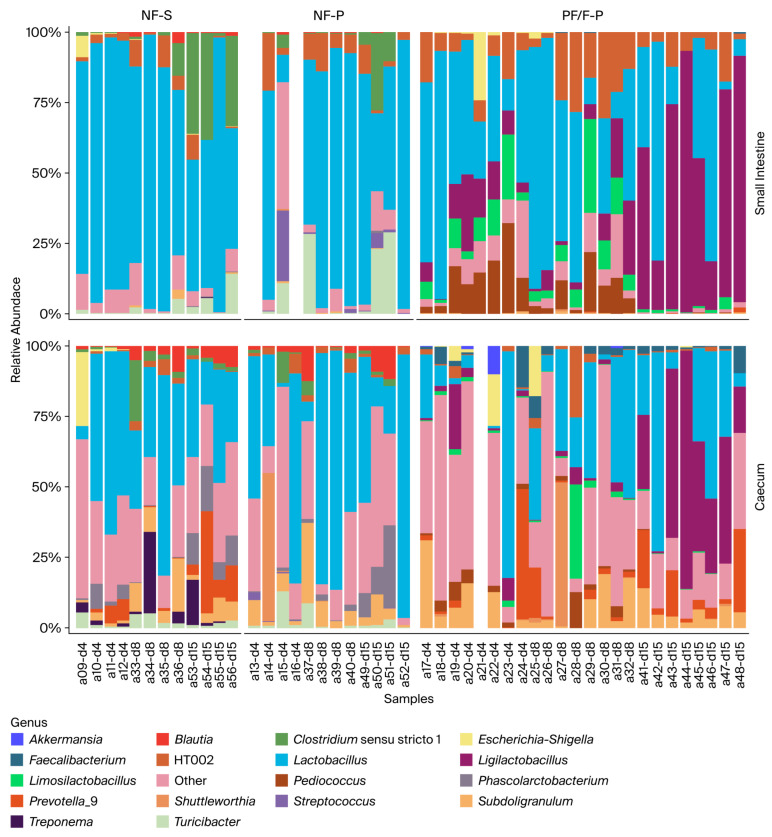
Relative genus-level composition of the small-intestinal and cecal microbiota in weaned piglets, with treatments containing the fermented pea–wheat ingredient combined for interpretation. Notes: NF-S: diet containing 6% non-fermented soybean meal; NF-P: diet in which all soybean meal was replaced by 12% non-fermented field peas; PF-P: diet in which 50% of the field pea fraction (6%) was supplied through the fermented pea–wheat ingredient; F-P: diet in which the full field pea fraction (12%) was supplied through the fermented pea–wheat ingredient.

**Figure 9:**
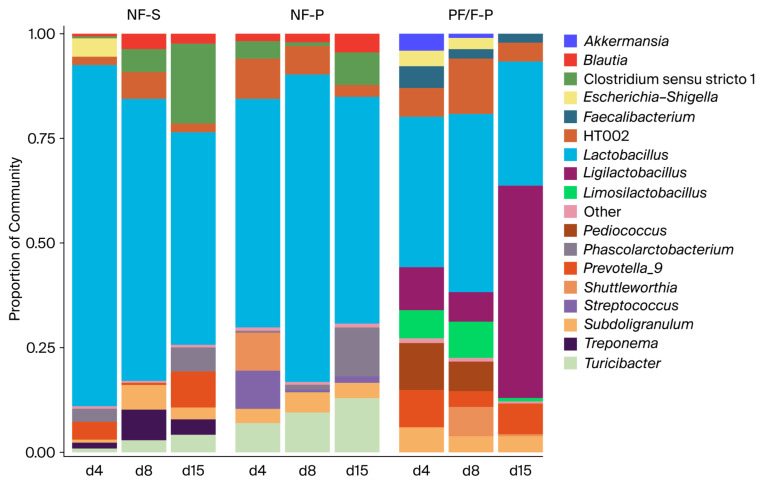
Average genus-level microbial community structure by treatment and sampling day in weaned piglets. Notes: NF-S: diet containing 6% non-fermented soybean meal; NF-P: diet in which all soybean meal was replaced by 12% non-fermented field peas; PF-P: diet in which 50% of the field pea fraction (6%) was supplied through the fermented pea–wheat ingredient; F-P: diet in which the full field pea fraction (12%) was supplied through the fermented pea–wheat ingredient.

Alpha diversity was assessed using two complementary indices, the Shannon index and the inverse Simpson index, which capture different aspects of microbial richness and evenness. Together, these metrics provided an overview of the effects of the dietary treatments on the microbiota of the small intestine and cecum in weaned piglets (Figure 7, Figure 8 and Figure 9).

In the non-fermented soybean-based control group (NF-S), both diversity indices showed a moderate increase or stabilization over time, particularly in the cecum. This pattern is consistent with the gradual diversification expected during normal post-weaning microbial colonization. In the non-fermented pea group (NF-P), diversity followed a similar trajectory; however, by day 15, the highest Shannon and inverse Simpson values were observed in the cecum of this group. This may reflect the provision of fermentable substrates from plant-derived proteins and fiber fractions, which could support the development of a more complex microbial community.

The diversity dynamics in piglets fed fermented pea–wheat diets differed from those of the non-fermented groups. On day 4, the inverse Simpson index was relatively high in the cecum, suggesting a transient increase in community evenness. Thereafter, both the Shannon and inverse Simpson indices declined, and by day 15, the PF-P and F-P groups showed lower diversity values than the non-fermented groups.

These findings suggest that diets containing the fermented pea–wheat ingredient were associated with changes in microbial diversity and relative taxon abundance over time. However, the biological implications of these diversity patterns remain uncertain and cannot be interpreted as beneficial or detrimental without additional functional data.

Taken together, these diversity data indicate that the non-fermented diets, particularly the pea-containing diet, were associated with gradual changes in microbial diversity and community structure, whereas diets containing the fermented pea–wheat ingredient were associated with distinct compositional changes following an early transient increase in diversity. This pattern is consistent with the possibility that fermented feeds influence the relative abundance of specific bacterial groups rather than increasing overall diversity.

#### 3.5.4. Changes in Beta Diversity (Bray–Curtis)

**Figure 10:**
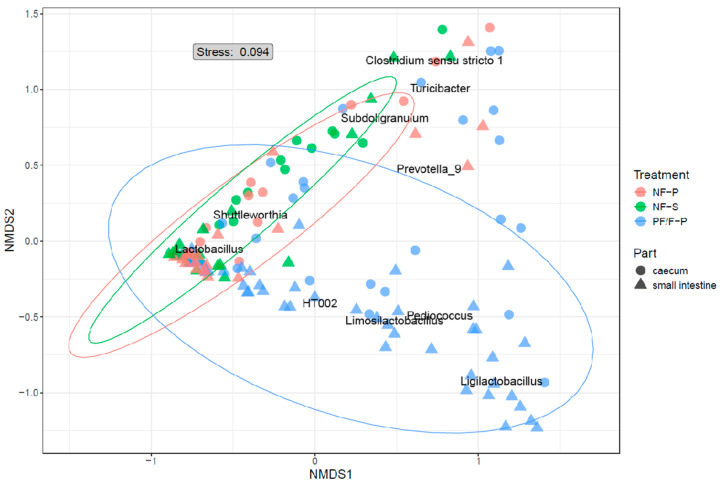
Nonmetric multidimensional scaling of gut microbial community structure based on Bray–Curtis dissimilarity in weaned piglets fed diets differing in fermentation status. Notes: NF-S: diet containing 6% non-fermented soybean meal; NF-P: diet in which all soybean meal was replaced by 12% non-fermented field peas; PF-P: diet in which 50% of the field pea fraction (6%) was supplied through the fermented pea–wheat ingredient; F-P: diet in which the full field pea fraction (12%) was supplied through the fermented pea–wheat ingredient.

Beta diversity was assessed by NMDS based on Bray–Curtis dissimilarity (stress = 0.094), indicating a good representation of the data in two-dimensional space. The ordination showed differences in microbial community structure among dietary treatments (Figure 10). These ordination patterns describe compositional differences only and should not be interpreted as evidence of functional divergence among microbial communities.

Samples from piglets receiving the two non-fermented diets (NF-S and NF-P) clustered separately from those of piglets receiving diets containing the fermented pea–wheat ingredient (PF-P and F-P). The NF-S and NF-P samples were located mainly in the negative range of the ordination axes and formed relatively compact clusters, indicating similar microbial community structures under the two non-fermented diets. In contrast, samples from the PF-P and F-P groups were located primarily in the positive range of the ordination and showed greater dispersion, suggesting treatment-associated differences in microbial composition in piglets receiving fermented feed.

These differences in community structure were consistent with the distribution of dominant taxa shown in Figure 10. *Lactobacillus* and *Shuttleworthia* were more closely associated with the non-fermented groups, whereas many PF-P and F-P samples were characterized by genera such as *Ligilactobacillus*, *Pediococcus*, *Limosilactobacillus*, and *Prevotella_9*. This pattern suggests compositional differences associated with diets containing the fermented pea–wheat ingredient. Accordingly, diets containing the fermented pea–wheat ingredient were associated with differences not only in alpha diversity patterns but also in overall microbial community structure.

Differences among intestinal regions were also evident. Cecal samples showed broader spatial dispersion, whereas small-intestinal samples formed more compact clusters, reflecting the distinct microecological environments of these regions.

Overall, the NMDS analysis indicated that diets containing the fermented pea–wheat ingredient were associated with variation in microbial community structure relative to the non-fermented diets. These differences were reflected in compositional variation and greater dispersion among samples. However, because the analysis is based on compositional data, the functional implications of these shifts cannot be determined from the present dataset.

## 4. Discussion

The post-weaning period represents a major physiological challenge for piglets and is characterized by abrupt dietary change, altered microbial exposure, and transient impairment of intestinal structure and function. In this study, pea-based liquid diets differing in the inclusion level of a fermented pea–wheat ingredient were associated with differences in intestinal morphology, epithelial proliferation, microbiota composition, and pen-level fecal consistency, whereas systemic inflammatory cytokine responses remained low across treatments.

Circulating concentrations of IL-1β, IL-6, and TNF-α remained extremely low across all dietary treatments and sampling days and were frequently close to or below the detection limits of the ELISA assays. No statistically significant differences were detected among piglets fed the NF-S, NF-P, PF-P, or F-P diets, indicating that none of the feeding strategies induced measurable systemic inflammation during the post-weaning period. This observation is consistent with previous reports showing that, under non-pathogenic conditions, weaning-associated inflammation is often localized primarily to the intestinal mucosa and does not necessarily result in elevated circulating cytokine concentrations unless piglets are exposed to severe enteric infection or endotoxin challenge. Several studies have reported that fermented protein ingredients can improve intestinal morphology and gut function in weaned piglets without inducing measurable systemic inflammatory responses [20,35,40,41]. Similar observations have also been reported under enteric challenge conditions, where fermented soybean meal alleviated diarrhea and intestinal inflammation without increasing circulating cytokine concentrations [22]. Together, these findings support the interpretation that the low systemic cytokine values observed in the present study are compatible with localized intestinal adaptation rather than systemic inflammatory activation.

The low systemic cytokine values occurred alongside differences in intestinal morphology and epithelial proliferation. Weaning is typically associated with villus atrophy and crypt hyperplasia during the first days after separation from the sow, driven by reduced feed intake, increased luminal antigen exposure, and inflammatory stimuli. In the present experiment, no morphological differences were observed on the day of weaning, which is consistent with the view that structural divergence develops only after dietary exposure. By day 4 after weaning, piglets fed diets containing the fermented pea–wheat ingredient had significantly greater villus height in the duodenum, jejunum, and ileum than piglets fed non-fermented diets. This finding may be biologically relevant because villus height commonly declines sharply within 72–96 h after weaning, although the functional significance of this response cannot be confirmed from the present data alone. Comparable protection against villus atrophy has been reported for fermented soybean meal and other fermented protein sources [20,40,42], indicating that fermentation may be compatible with preservation of mucosal architecture during the early phase of post-weaning stress.

The morphological differences associated with diets containing the fermented pea–wheat ingredient may reflect multiple complementary effects of fermentation. Fermentation can reduce antinutritional compounds, including trypsin inhibitors and lectins, while generating organic acids and other metabolites that may influence intestinal conditions and nutrient utilization [41,43]. Previous studies have similarly reported improved villus architecture and modulation of intestinal microbiota in piglets receiving fermented protein ingredients or pea-based diets [35,44]. In the present study, higher villus-to-crypt ratios during the early post-weaning phase occurred alongside lower pen-level fecal scores in the pen assigned to the F-P diet, although direct functional relationships cannot be established from the present data. As post-weaning adaptation progressed, treatment effects on villus height became increasingly segment-specific, likely reflecting regional differences in nutrient flow, fermentation activity, and microbial composition. By day 8, between-treatment variation was especially evident in the jejunum and ileum, consistent with previous findings that peas and pea-derived fractions can exert stronger effects in distal regions of the small intestine [44,45]. By day 15, villus height had partially converged across treatments, reflecting the expected recovery that occurs as feed intake stabilizes. Nevertheless, diets containing the fermented pea–wheat ingredient remained associated with greater villus height in selected intestinal segments, in agreement with previous reports describing morphological responses to fermented rapeseed, soybean products, and lactobacilli-based diets [35,46].

Changes in intestinal morphology were paralleled by alterations in epithelial proliferative activity, as indicated by Ki-67 immunostaining. Under physiological conditions, the proportion of Ki-67-positive cells in weaned piglets typically ranges from 30% to 50%, reflecting the high epithelial turnover required to maintain intestinal integrity [47]. However, weaning stress can suppress Ki-67 expression during the first few days after weaning because of inflammation, villus atrophy, and reduced nutrient intake. In the present study, partial inclusion of the fermented pea–wheat ingredient was associated with a marked increase in crypt cell proliferation by day 4, suggesting rapid compensatory epithelial renewal during the acute stress phase. In contrast, full inclusion of the fermented pea–wheat ingredient was associated with lower initial Ki-67 indices, which may indicate earlier mucosal stabilization and a reduced need for hyperproliferation. By day 8, the F-P diet was associated with the highest Ki-67 values in the duodenum and jejunum, coinciding with enrichment of *Ligilactobacillus* and *Limosilactobacillus*. Previous studies have associated these genera with epithelial regeneration and intestinal barrier function [48,49,50]. By day 15, proliferative activity had largely converged across treatments, suggesting progressive mucosal recovery during the post-weaning period.

The morphological and proliferative responses observed in this study were accompanied by differences in microbiota composition. Diets containing the fermented pea–wheat ingredient were associated with higher relative abundance of lactic acid-related genera such as *Ligilactobacillus*, *Limosilactobacillus*, and *Lactobacillus sensu stricto* across intestinal segments. These shifts resemble microbial patterns reported in piglets receiving probiotic supplementation or fermented soybean meal [51,52]. The predominance of *Ligilactobacillus salivarius*, together with the increased abundance of *Limosilactobacillus reuteri* and *Ligilactobacillus murinus*, may be associated with mechanisms related to immune regulation, epithelial function, and barrier integrity [53,54,55]. However, these interpretations remain hypothesis-generating, as the functional relevance of these taxa cannot be confirmed from 16S rRNA compositional data alone. In addition, the greater relative abundance of *Prevotella_9* in the large intestine may be consistent with compositional patterns often associated with plant-polysaccharide fermentation. Members of this genus have been linked to short-chain fatty acid production, which may influence epithelial function and immune responses [51,52]. These microbial shifts occurred in parallel with differences in fecal consistency; however, causal relationships cannot be established from the present study.

Another aspect that warrants consideration is the relatively low α-diversity observed on day 15 in the groups receiving diets containing the fermented pea–wheat ingredient, together with the pronounced dominance of lactobacilli. Although reduced microbial diversity is often interpreted negatively, in the present nutritional context it may reflect a more selective microbial composition. However, its functional implications cannot be determined from the present data alone. Restricted feeding and reduced dietary crude protein may also have contributed to the microbiota patterns observed in the present study by limiting the amount of undigested protein reaching the hindgut [56,57,58]. Under such conditions, microbial communities may shift toward taxa associated with carbohydrate fermentation rather than proteolytic fermentation. Similar associations between lower α-diversity and improved gut-related parameters have previously been reported in piglets receiving highly digestible or fermented protein sources [59,60,61]. Therefore, the dominance of *Lactobacillus* and related genera in groups receiving diets containing the fermented pea–wheat ingredient may indicate a shift toward microbial taxa commonly associated with carbohydrate fermentation and organic acid production, although this potential functional interpretation requires confirmation through metabolomic or functional microbiome analyses.

When interpreting these results, the feeding strategy applied in the present experiment should also be considered. Feed intake during the early post-weaning period strongly influences intestinal morphology, microbial colonization, and growth performance. For example, Engelsmann et al. [62] reported that increased feed intake in the days following weaning can enhance growth performance but may also increase the risk of post-weaning diarrhea and subsequent antibiotic use. In the present study, a restricted feeding regimen was used to minimize variation associated with voluntary feed intake, allowing a more controlled assessment of gastrointestinal responses to dietary treatments. Therefore, the observed effects should primarily be interpreted in the context of intestinal adaptation under controlled feeding conditions rather than as a direct reflection of ad libitum feeding systems used in commercial production. Taken together, the present findings indicate that diets containing the fermented pea–wheat ingredient were associated with differences in intestinal morphology, epithelial proliferation, microbiota composition, and pen-level fecal consistency during the early post-weaning period. However, because the study was performed under controlled feeding conditions and relied primarily on compositional microbiota data, the functional significance and mechanistic basis of these responses remain to be clarified in future studies.

Several limitations should be acknowledged. First, several nutritional and management factors were applied simultaneously, including reduced crude protein levels, fiber inclusion, restricted feeding, and the use of fermented ingredients. Consequently, the observed responses should be interpreted as the combined effect of the overall dietary system, and specific outcomes cannot be attributed exclusively to the inclusion of the fermented pea–wheat ingredient. In addition, the fermented pea–wheat ingredient was characterized in the present study only in terms of dry matter, pH, and selected microbial counts. Organic acid profiles, including lactic and acetic acid concentrations, and residual antinutritional factors were not determined in this animal experiment. Therefore, the present data cannot directly link fermentation-induced biochemical changes in the ingredient to the observed gastrointestinal responses. Future studies should include detailed biochemical characterization of the fermented ingredient, including organic acids, residual antinutritional compounds, and relevant fermentation metabolites. Second, the sequential sampling design resulted in relatively small sample sizes at each time point, which may have limited statistical power and reduced the ability to detect more subtle treatment effects. In addition, multiple comparisons were performed across treatments, intestinal segments, sampling days, and outcome variables. Although Tukey’s post hoc test and appropriate non-parametric tests were applied within individual analyses, the cumulative risk of type I error across the overall analytical framework cannot be excluded. Most importantly, the dietary treatments were not replicated at the pen level. The pen was the experimental unit for dietary treatment, whereas individual animals provided analytical units for the endpoints measured on individual samples. Therefore, treatment-associated differences reported in this study should be interpreted as exploratory and hypothesis-generating, and the possibility of pen effects cannot be excluded. Third, the study was restricted to the early post-weaning period and therefore cannot establish whether the observed effects persist during later production stages. Finally, the inflammatory assessment was based primarily on circulating cytokines and may not have captured localized mucosal immune responses. Future studies should combine longer-term validation with tissue-level analyses of immune function and barrier integrity, together with metabolomic approaches, to better define the mechanistic links among fermented pea–wheat-based diets, microbial activity, and host intestinal adaptation. Confirmation under commercial production conditions will also be important for determining broader translational relevance.

## 5. Conclusions

Under the controlled conditions of this short-term post-weaning study, diets containing the fermented pea–wheat ingredient were associated with differences in intestinal morphology, epithelial proliferation, microbiota composition, and pen-level fecal consistency. Full inclusion of the fermented pea–wheat ingredient was associated with consistently low fecal scores at pen level, whereas partial inclusion showed higher descriptive fecal scores during the study period. Because dietary treatment and pen were confounded and several nutritional and management factors were applied simultaneously, these findings should be interpreted as associations within the tested dietary system rather than as evidence of isolated effects of fermentation. Further studies using replicated pen designs, longer-term performance assessment, and detailed biochemical and functional analyses are required before practical recommendations can be made.

## Figures and Tables

**Table 1:** Composition of experimental diets (as-fed basis, g/kg).

	Dietary Treatment							
Item	NF-S		NF-P		PF-P		F-P	
	As-Fed Basis	88% Dry Matter Basis	As-Fed Basis	88% Dry Matter Basis	As-Fed Basis	88% Dry Matter Basis	As-Fed Basis	88% Dry Matter Basis
Ingredient, g/kg:								
Water	714.3	-	714.3	-	660.7	-	607.2	-
Wheat	118.8	415.8	101.1	354	95.4	334	89.7	314
Barley	98.4	345	98.4	344.5	98.4	344.5	98.4	344.5
AlphaSoy 530 ^1^	24	84	24	84	24	84	24	84
Soybean meal 46	17.2	60	-	-	-	-	-	-
Peas—unfermented	-	-	34.3	120	17.2	60	-	-
F-75P25W	-	-	-	-	76.4		152.8	
Peas—fermented	-	-	-	-	(17.2)	60	(34.3)	120
Wheat—fermented	-	-	-	-	(5.7)	20	(11.4)	40
Lactomel NG ^2^	14.4	50	14.4	50.4	14.4	50.4	14.4	50.4
Arbocel RC fine ^3^	5.8	20	5.8	20.3	5.8	20.3	5.8	20.3
Salt	1.7	6	1.7	6	1.7	6	1.7	6
Piglet premix 0.5% ^4^	1.4	5	1.4	5	1.4	5	1.4	5
Ca-formate ^5^	1.2	4.2	1.2	4.2	1.2	4.2	1.2	4.2
Lysine-HCl 79% ^5^	1.2	4.2	1.3	4.2	1.3	4.2	1.3	4.2
Limestone	0.7	2.5	0.7	2.5	0.7	2.5	0.7	2.5
Threonine 98% ^5^	0.5	1.8	0.6	2.1	0.6	2.1	0.6	2.1
Methionine 99% ^6^	0.3	1.1	0.4	1.4	0.4	1.4	0.4	1.4
Tryptophan 98% ^7^	0.1	0.4	0.2	0.7	0.2	0.7	0.2	0.7
Valine 99% ^7^	-	-	0.2	0.7	0.2	0.7	0.2	0.7
Nutrient and energy composition (g/kg, MJ/kg):								
Dry matter, g	254	880	254	880	254	880	254	880
ME—swine, MJ	3.9	13.7	3.9	13.7	3.9	13.7	3.9	13.7
NE—swine, MJ	2.8	9.7	2.8	9.8	2.8	9.8	2.8	9.8
Crude protein, g	47	164	45	154	45	154	45	154
Crude fat, g	6.0	21	6.0	20	6.0	20	6.0	20
Crude fiber, g	14	48	14	48	14	48	14	48
Ash, g	12	41	12	34	12	40	12	40
Starch, g	127	439	132	458	133	458	133	458
Sugar, g	18	61	17	59	17	59	17	59
Lysine, g	0.3	1.1	0.3	1.1	0.3	1.1	0.3	1.1
SID lysine, g	2.7	9.4	2.8	9.5	2.8	9.5	2.8	9.5

Abbreviations and notes: NF-S: diet containing 6% non-fermented soybean meal; NF-P: diet in which all soybean meal was replaced by 12% non-fermented field peas; PF-P: diet in which 50% of the field pea fraction (6%) was supplied through the fermented pea–wheat ingredient; F-P: diet in which the full field pea fraction (12%) was supplied through the fermented pea–wheat ingredient. F-75P25W: fermented feed ingredient with a dry matter content of 26.3%; ME: metabolizable energy; NE: net energy; SID lysine: standardized ileal digestible lysine. Producers/distributors: ^1^ AB Neo (Videbaek, Denmark); ^2^ Schils BV (Sittard, Netherlands); ^3^ Rettenmaier Austria GmbH & Co. (Wien, Austria); ^4^ Agrofeed Ltd. (Győr, Hungary); ^5^ AB Kauno Grūdai (Kaunas, Lithuania); ^6^ Evonik Operations GmbH (Essen, Germany); ^7^ CJ Europe GmbH (Schwalbach, Germany).

**Table 2:** Daily dry and liquid feeding schedule of experimental weaned piglets (kg/head).

Day	Air-Dry Feed Allowance	Liquid Diet
1	0.200	0.70
2	0.225	0.79
3 *	0.300	1.05
4 *	0.350	1.23
5	0.375	1.31
6	0.400	1.40
7	0.425	1.49
8	0.450	1.58
9	0.475	1.66
10	0.500	1.75
11	0.525	1.84
12	0.550	1.93
13	0.575	2.01
14	0.600	2.10
15	0.150	0.53
Total	6.100	21.35

* Additional adjustment to compensate for appetite.

**Table 3:** Daily pen-level fecal consistency scores by dietary treatment.

Day	NF-S	NF-P	PF-P	F-P
1	0	0	0	0
2	0	0	0	0
3	0	0	0	0
4	0	0	1	0
5	0	0	1	0
6	0	1	1	0
7	0	2	1	0
8	0	0	0	0
9	0	0	0	0
10	0	0	0	0
11	1	0	2	0
12	0	2	2	0
13	0	1	1	0
14	0	1	2	0
15	0	0	2	0
Average	0.07	0.47	0.87	0.00

Notes: NF-S: diet containing 6% non-fermented soybean meal; NF-P: diet in which all soybean meal was replaced by 12% non-fermented field peas; PF-P: diet in which 50% of the field pea fraction (6%) was supplied through the fermented pea–wheat ingredient; F-P: diet in which the full field pea fraction (12%) was supplied through the fermented pea–wheat ingredient.

**Table 4:** Villus height (VH), crypt depth (CD), and villus height-to-crypt depth (VH:CD) ratio in the duodenum, jejunum, and ileum of weaned piglets.

Day	Intestinal Region	Morphology	NF-S	NF-P	PF-P	F-P	SEM	*p*-Value
0	Duodenum	VH (µm)	354.174	352.278	341.201	359.317	-	-
		CD (µm)	185.573	181.152	187.643	181.960	-	-
		VH:CD ratio	1.951	1.950	1.839	1.983	-	-
	Jejunum	VH (µm)	350.963	350.310	350.762	348.216	-	-
		CD (µm)	149.822	149.527	154.806	154.902	-	-
		VH:CD ratio	2.536	2.404	2.364	2.379	-	-
	Ileum	VH (µm)	327.369	332.367	323.522	334.140	-	-
		CD (µm)	143.168	134.456	140.731	138.323	-	-
		VH:CD ratio	2.375	2.554	2.457	2.470	-	-
4	Duodenum	VH (µm)	385.539 b	388.841 b	432.901 a	441.693 a	4.865	0.001
		CD (µm)	199.674 b	182.632 a	196.663 b	207.195 b	3.357	0.001
		VH:CD ratio	2.028 b	2.160 ab	2.251 a	2.158 ab	0.041	0.002
	Jejunum	VH (µm)	371.759 b	377.576 b	428.813 a	427.592 a	4.405	0.001
		CD (µm)	165.741 a	177.161 b	200.264 c	198.146 c	2.819	0.001
		VH:CD ratio	2.309 a	2.164 b	2.165 b	2.188 ab	0.036	0.013
	Ileum	VH (µm)	342.607 b	348.892 b	398.643 a	389.391 a	4.371	0.001
		CD (µm)	154.637 a	164.895 a	180.285 b	186.330 b	3.159	0.001
		VH:CD ratio	2.321	2.183	2.280	2.142	0.052	0.051
8	Duodenum	VH (µm)	383.997 b	393.685 b	474.310 a	482.856 a	5.439	0.001
		CD (µm)	182.374 a	169.446 a	258.711 c	212.668 b	3.811	0.001
		VH:CD ratio	2.161 b	2.417 a	1.892 c	2.338 ab	0.049	0.001
	Jejunum	VH (µm)	375.183 ab	384.318 a	355.048 b	393.901 a	5.967	0.001
		CD (µm)	166.424 a	184.864 b	162.578 a	165.909 a	3.277	0.001
		VH:CD ratio	2.357 a	2.107 b	2.282 ab	2.462 a	0.060	0.001
	Ileum	VH (µm)	338.691 b	361.926 a	339.981 b	342.037 b	5.077	0.003
		CD (µm)	143.402 a	166.591 b	148.487 a	151.387 a	3.668	0.001
		VH:CD ratio	2.477	2.246	2.447	2.399	0.069	0.082
15	Duodenum	VH (µm)	411.927 b	414.778 b	445.932 a	423.852 ab	7.216	0.004
		CD (µm)	231.022	221.618	226.268	216.873	5.862	0.359
		VH:CD ratio	1.935	2.050	2.138	2.120	0.081	0.283
	Jejunum	VH (µm)	422.008 a	419.553 a	394.025 b	414.024 ab	7.753	0.047
		CD (µm)	174.098 bc	142.010 a	163.514 b	180.381 c	3.981	0.001
		VH:CD ratio	2.483 b	3.216 a	2.602 b	2.433 b	0.093	0.001
	Ileum	VH (µm)	317.633 b	328.603 b	384.724 a	338.147 b	6.487	0.001
		CD (µm)	129.537 a	139.681 ab	158.729 c	143.269 b	3.649	0.001
		VH:CD ratio	2.677	2.518	2.557	2.483	0.087	0.417

Notes: NF-S: diet containing 6% non-fermented soybean meal; NF-P: diet in which all soybean meal was replaced by 12% non-fermented field peas; PF-P: diet in which 50% of the field pea fraction (6%) was supplied through the fermented pea–wheat ingredient; F-P: diet in which the full field pea fraction (12%) was supplied through the fermented pea–wheat ingredient; a–c: different lowercase letters within the same row indicate significant differences at *p* < 0.05.

## Data Availability

The data presented in this study are available from the corresponding author upon reasonable request.

## Supplementary Materials

The following supporting information can be downloaded at: https://www.mdpi.com/article/10.3390/ani16101526/s1, Figure S1: Plasma absorbance values for interleukin-1β. Notes: NF-S: diet containing 6% non-fermented soybean meal; NF-P: diet in which all soybean meal was replaced by 12% non-fermented field peas; PF-P: diet in which 50% of the field pea fraction (6%) was supplied through the fermented pea–wheat ingredient; F-P: diet in which the full field pea fraction (12%) was supplied through the fermented pea–wheat ingredient; Figure S2: Plasma absorbance values for interleukin-6. Notes: NF-S: diet containing 6% non-fermented soybean meal; NF-P: diet in which all soybean meal was replaced by 12% non-fermented field peas; PF-P: diet in which 50% of the field pea fraction (6%) was supplied through the fermented pea–wheat ingredient; F-P: diet in which the full field pea fraction (12%) was supplied through the fermented pea–wheat ingredient; Figure S3: Plasma absorbance values for tumor necrosis factor-α. Notes: NF-S: diet containing 6% non-fermented soybean meal; NF-P: diet in which all soybean meal was replaced by 12% non-fermented field peas; PF-P: diet in which 50% of the field pea fraction (6%) was supplied through the fermented pea–wheat ingredient; F-P: diet in which the full field pea fraction (12%) was supplied through the fermented pea–wheat ingredient.
