# Supplementary material for: Effects of Fermented Pea–Wheat Ingredient Inclusion in Soybean Meal-Replacement Diets on Intestinal Adaptation, Gut Microbiota, and Fecal Consistency in Weaned Piglets
Source: Animals (Basel). 2026 May 16;16(10):1526. doi: 10.3390/ani16101526 (PMC13203612; doi:10.3390/ani16101526)
Supplement: Supplementary file 1 [file animals-16-01526-s001.zip › animals-4264277-supplementary.pdf]

Supplementary Materials

Effects of Fermented Pea–Wheat Ingredient Inclusion in Soybean Meal-Replacement Diets on Intestinal Adaptation, Gut Microbiota, and Fecal Consistency in Weaned Piglets

Botond Alpár, László Varga, Alex Váradi, Eszter Kaszab, Zoltán Somogyi and Tamás Tóth

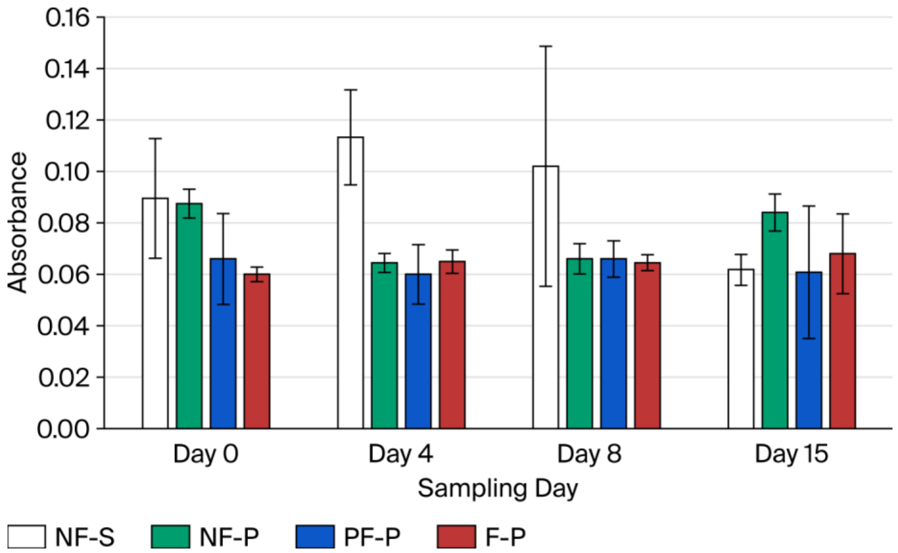

Figure S1. Plasma absorbance values for interleukin-1β. Notes: NF-S: diet containing 6% non-fermented soybean meal; NF-P: diet in which all soybean meal was replaced by 12% non-fermented field peas; PF-P: diet in which 50% of the field pea fraction (6%) was supplied through the fermented pea–wheat ingredient; F-P: diet in which the full field pea fraction (12%) was supplied through the fermented pea–wheat ingredient.

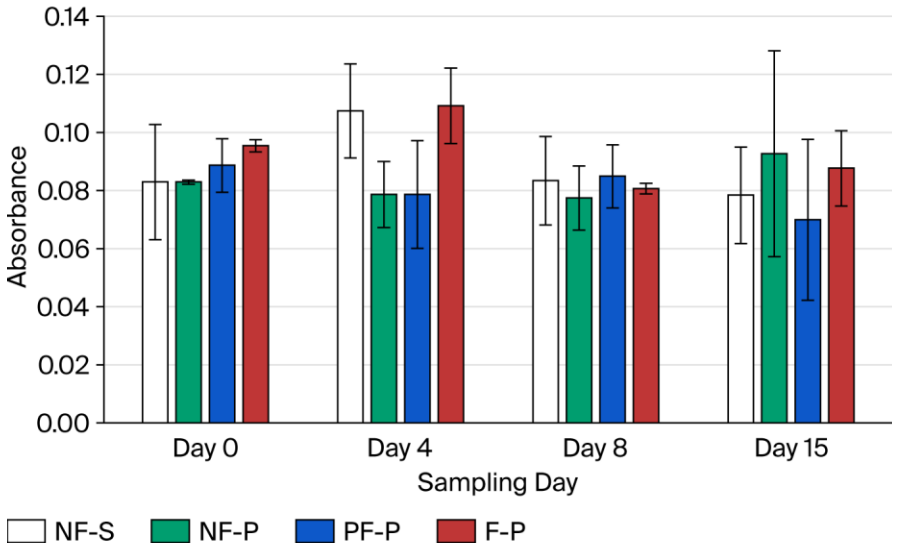

Figure S2. Plasma absorbance values for interleukin-6. Notes: NF-S: diet containing 6% non-fermented soybean meal; NF-P: diet in which all soybean meal was replaced by 12% non-fermented field peas; PF-P: diet in which 50% of the field pea fraction (6%) was supplied through the fermented pea–wheat ingredient; F-P: diet in which the full field pea fraction (12%) was supplied through the fermented pea–wheat ingredient.

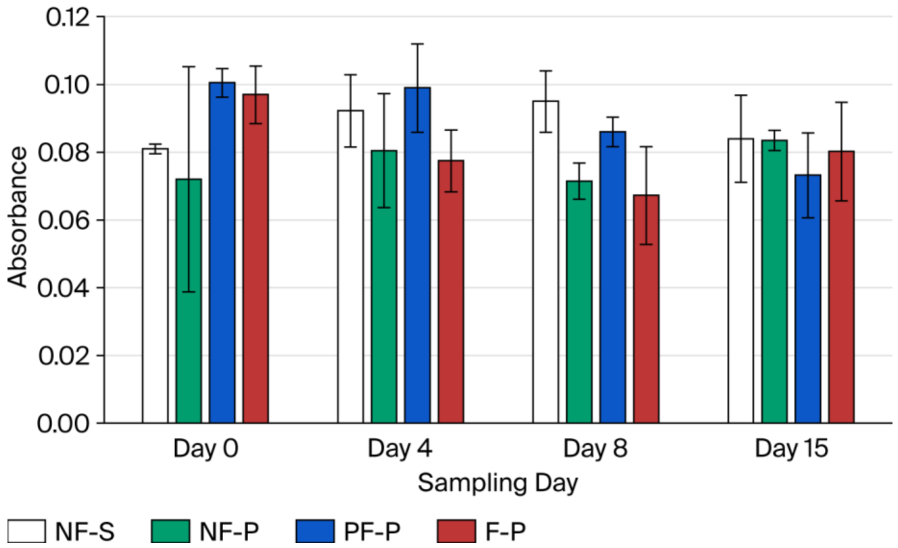

Figure S3. Plasma absorbance values for tumor necrosis factor-α. Notes: NF-S: diet containing 6% non-fermented soybean meal; NF-P: diet in which all soybean meal was replaced by 12% non-fermented field peas; PF-P: diet in which 50% of the field pea fraction (6%) was supplied through the fermented pea–wheat ingredient; F-P: diet in which the full field pea fraction (12%) was supplied through the fermented pea–wheat ingredient.
